# Antinociceptive activities of *Artocarpus lacucha* Buch-ham (Moraceae) and its isolated phenolic compound, catechin, in mice

**DOI:** 10.1186/s12906-019-2565-x

**Published:** 2019-08-14

**Authors:** Shanta Islam, Md. Shafiullah Shajib, Ridwan Bin Rashid, Mohammad Firoz Khan, Md. Abdullah Al-Mansur, Bidyut Kanti Datta, Mohammad Abdur Rashid

**Affiliations:** 1 Department of Pharmacy, Stamford University Bangladesh, 51 Siddeswari Road, Dhaka, 1217 Bangladesh; 2 Department of Pharmacy, State University of Bangladesh, Dhaka, 1205 Bangladesh; 3 Institute of National Analytical Research and Service (INARS), Bangladesh Council of Scientific and Industrial Research (BCSIR), Dhaka, Bangladesh; 4 Department of Pharmaceutical Chemistry, Faculty of Pharmacy, University of Dhaka, Dhaka, 1000 Bangladesh

**Keywords:** *Artocarpus lacucha*, Traditional system of medicine, Phenolic, (+)-catechin, Opioid, ATP-sensitive K^+^ channel

## Abstract

**Background:**

The present study evaluated the antinociceptive effect of the bark of *Artocarpus lacucha*, which is used for the treatment of stomachache, headache and boils in the traditional system of medicine.

**Methods:**

The antinociceptive activity was investigated by the tail immersion, hot plate, acetic acid- & formalin-induced nociception and carrageenan-induced paw edema tests using a hydro-methanolic extract of *A. lacucha* bark. The plant extract was found to contain a substantial amount of phenolic compounds according to the total phenolic and flavonoid content assay. A phenolic metabolite, (+)-catechin, has been isolated using different chromatographic techniques. The compound was characterized with 1D and 2D NMR spectroscopic data. (+)-catechin, isolated from *A. lacucha* was assessed for antinociceptive effects swiss albino mice. Furthermore, the possible involvement of opioid receptors and ATP-sensitive K^+^ channel for the effect of the plant extract and (+)-catechin has been justified using naloxone and glibenclamide, respectively.

**Results:**

Oral administration (p.o) of the plant extract (50–200 mg/Kg b.w.) resulted in significant thermal pain protection in the hot plate and tail immersion tests. The action of the plant extract was significantly antagonized by naloxone, a non-selective opioid antagonist, in the hot plate and tail immersion tests, which supports the involvement of opioid receptors. Both the plant extract and (+)-catechin, (50–200 mg/Kg b.w., p.o.) significantly diminished the acetic acid- & formalin-induced nociception, and carrageenan-induced paw edema. Glibenclamide, an ATP-sensitive K^+^ channel blocker, significantly reversed their effect in the acetic acid-induced writhing test which indicates the participation of ATP-sensitive K^+^ channel system.

**Conclusions:**

The investigation revealed potential central and peripheral antinociceptive effects of *A. lacucha* bark supports its applications in the traditional system of medicine.

**Electronic supplementary material:**

The online version of this article (10.1186/s12906-019-2565-x) contains supplementary material, which is available to authorized users.

## Background

The nociception of central and peripheral nervous systems can lead to the development and persistence of the pathological conditions of pain [1, 2]. Analgesic therapeutics including glucocorticoids, non-steroidal anti-inflammatory drugs, opioids are currently available but their significant detrimental effects have thwarted their applications [3–5]. Over the last few years, after countless investigations, researchers found that natural products derived from medicinal plants could be effective in analgesic therapy with a wider safety margin, high efficiency and fewer side effects/adverse effects [6, 7]. The medicinal plants of the genus, *Artocarpus*, belongs to the Moraceae family and comprises of about 50 species. It has been reported that both crude extracts and isolated phenolic compounds from *Artocarpus* species are known to possess promising anti-arthritic, anti-inflammatory and antioxidant activities [8]. *Artocarpus lacucha* Buch-Ham (Moraceae) is a deciduous tree, which is planted all over Bangladesh for its edible fruits. It is called Monkey jack and locally known as Deowa [9]. In traditional systems of medicine, the plant is used for anti-inflammatory therapy. The bark of the plant is used to treat stomachache, headache, fever and liver diseases [10–12]. The bark powder is applied externally on boils, pimples, cracked skin and as an antiseptic [13].

It has been reported that the methanol extract of the bark of *A. lacucha* contains considerable amounts of phenolics and flavonoids [14]. The tree bark has also been reported to contain amyrin acetate, lupeol acetate, 5-(*γ*,*γ* -dimethylallyl)-oxyresveratrol, 3-(*γ*,*γ*-dimethylallyl)resveratrol, 3-(2,3-dihydroxy-3-methylbutyl)resveratrol, 3-(*γ*,*γ*-dimethylpropenyl)moracin M, afzelechin-3-*O*-R-L-rhamnopyranoside, oxyresveratrol, dihydromorin, (−)-epiafzelechin, and epiafzelechin-(4β → 8)-epicatechin [12, 15]. Trans-2,4,3′,5′-tetrahydroxystilbene (THS), a phenolic compound, isolated from an aqueous extract of the plant, has been found to possess anti-inflammatory, antioxidant, anti-HIV and antiherpetic activities [16]. The anti-inflammatory activity of the plant is evident from previous pharmacological reports [17]. Bark extract of the plant has been found to possess strong antioxidant activity [14]. Catechin, a phenolic compound of the bark and twigs of the plant, exhibited in-vitro cyclooxygenases (COX-1 and COX-2) inhibitory effects [15]. The promising in-vivo anti-arthritic activity of catechin has been previously reported [18]. Based on these findings, the present investigation was designed to evaluate the antinociceptive activity of a hydro-alcoholic extract of the bark of *A. lacucha* and its isolated compound, catechin, in experimental models of mice. Furthermore, the possible mechanism of the action of the bark extract and its isolate via opioid systems have been investigated in this study. It has been reported that activation of ATP sensitive K+ channel significantly modulate the sensory neurons excitability, thus on sensory behavior or nociception, Besides, it is evidenced that opioid and non-opioid analgesic may exert their antinociceptive effects via activation of this ion channel [19]. Therefore, the present study also evaluated the involvement of ATP sensitive K+ channel in the antinociceptive effect of HEBA and (+)-catechin.

## Materials and methods

### Plant material

*A. lacucha* was collected from Sonargoan, Narrayangonj, Bangladesh in October 2015. The plant materials including bark were authenticated by Mr. Ahsan Habib, Senior Scientific Officer, Bangladesh National Herbarium, Mirpur, Dhaka. A voucher specimen of the plant material was deposited in the herbarium under the number, DACB: 42083, for future reference. The bark of the plant was shade dried and grounded to a coarse powder. One kilogram of the bark powder was mixed with 4000 mL methanol-water (80:20) for 10 days. The extract was filtered using cotton bed followed by filter paper (Whatman No. 1) and the filtrate was concentrated using a rotary evaporator at 40 °C and 50 r.p.m. The concentrated filtrate was subjected to lyophilization after freezing. Finally, 82 g of dried extract (yield 8.20%) was obtained.

### Preliminary phytochemical screening

The plant extract was subjected to qualitative phytochemical screening for the detection of carbohydrates, glycosides, saponins, flavonoids, tannins, alkaloids, resins and steroids, by the methods described by Ghani (2003) [9]. Total flavonoid and phenolic contents of the extract were determined by using aluminum chloride (AlCl_3_) and Folin–Ciocalteu’s reagent according to the procedure described by Selim et al. (2014) [20] and Singleton et al. (1999) [21] respectively.

### Isolation and identification of compound

**Fig. 1:**
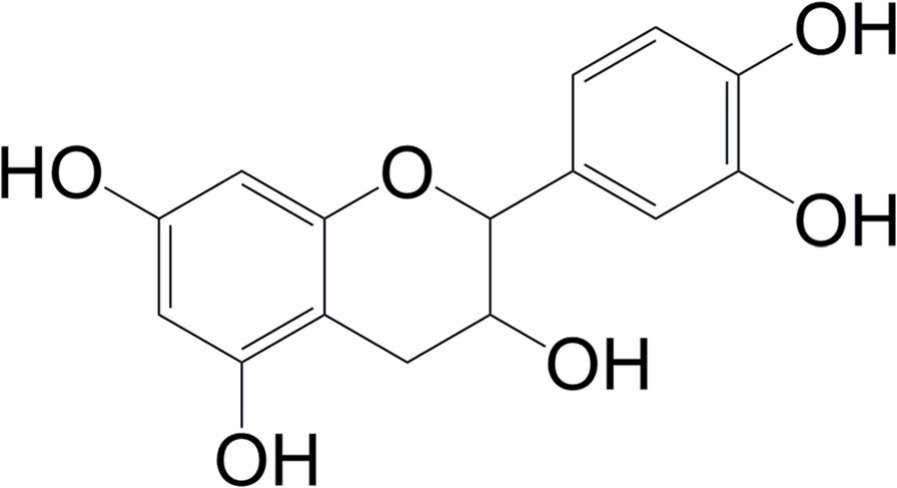
Structure of (+)-catechin isolated from a hydro-methanolic extract of the bark of *A. lacucha*

Thirty-five grams of bark extract was subjected to fractionation by Kieselgel (60–120 mesh, 710 g) vacuum liquid chromatography (VLC) using n-hexane, ethyl acetate (EtOAc) and methanol (MeOH). The collected fractions (41 fractions, 150 mL each) were analyzed by thin layer chromatography (TLC) and the similar fractions were combined together into five fractions (Fraction 1–5). After analysis the of combined fractions by TLC, fraction 2 (93% EtOAc in MeOH, 14.74 mg) was subjected to preparative thin layer chromatography over 60 F254, 20 × 20 cm PTLC plate which was developed with CHCl_3_: MeOH (4:1). The developed bands on the TLC plate was observed under UV light (254 nm and 356 nm) and was sprayed with 1% vanillin-sulfuric acid followed by heating at 110 °C. The selective band, with a R_f_ value of 0.15, was separated from the plate, eluted with ethyl acetate-chloroform (1:1), and chloroform-methanol (1:1). The solvents were dried to yield a pure compound (8 mg). The structure of the compound (Fig. 1) was elucidated as (+)-catechin by comprehensive analysis of ^1^H, ^13^C, DEPT, COSY, HSQC and HMBC NMR spectra (Additional file 1) and was confirmed by comparing the respective published spectral data [22, 23].

(+)-Catechin: pale yellow powder, m.p. 174–76 °C. ^1^H NMR (400 MHz, CD_3_OD): *δ*_H_ = 4.56 (1H, d; *J* = 7.60 Hz; H-2), 3.97 (1H, ddd; *J* = 5.20, 8.00, 7.60 Hz; H-3), 2.86 (1H, dd; *J* = 5.20, 16.00 Hz; H-4α), 2.52 (1H, dd; *J* = 8.00, 16.00 Hz; H-4β), 5.92 (1H, d; *J* = 1.80 Hz; H-6), 5.85 (1H, d; *J* = 1.80 Hz; H-8), 6.83 (1H, d; *J* = 1.20 Hz; H-2′), 6.76 (1H, d; *J* = 8.00 Hz; H-5′) and 6.72 (1H, dd; *J* = 8.00, 1.20 Hz; H-6′). ^13^C NMR (100 MHz, CD_3_OD): *δ*_C_ = 81.5 (C-2), 67.4 (C-3), 27.1 (C-4), 156.2 (C-5), 94.9 (C-6), 156.5 (C-7), 94.2 (C-8), 155.5 (C-9), 99.5 (C-10), 130.9 (C-1′), 113.9 (C-2′), 144.9 (C-3′), 144.9 (C-4′), 114.7 (C-5′) and 118.7 (C-6′).

### Chemicals and standard drugs

Methanol, chloroform, n-hexane, ethyl acetate, toluene, vanillin, sulfuric acid, acetic acid, and formalin were purchased from Merck Co. (Darmstadt, Germany). Methanol-d_4_ (CD_3_OD), sodium carboxymethylcellulose (Na-CMC), λ-carrageenan, (+)-catechin and pentobarbital sodium were purchased from Sigma-Aldrich (St. Louis, MO, USA). The standard drugs including, morphine sulfate (Gonoshasthaya Pharmaceuticals Ltd., Bangladesh), diclofenac sodium (Novartis Bangladesh Ltd., Bangladesh), naloxone hydrochloride (Samarth Life Sciences Pvt. Ltd., India), glibenclamide, diazepam (Square Pharmaceuticals Ltd., Bangladesh) were either purchased or was obtained as gifts.

### Animals

The experimental animals, Swiss albino male mice (4–6 weeks old, 20–25 g), were procured from Animal Resources Branch of the International Center for Diarrheal Disease Research, Bangladesh (icddr,b). The animals were housed in wooden cages (120 × 30 × 30 cm) containing flake wood shavings as bedding. All the animals were kept under standard laboratory environmental condition with 12 h light/dark cycle (lights on at 6.00 a.m. and off at 6.00 p.m.), 25 ± 2 °C room temperature and 55–60% relative humidity. They were fed with standard pellet diet manufactured by icddr,b, and access was given to tap water ad libitum. For acclimatization, the animals were left for 14 days in the laboratory. Health status of every mouse was checked regularly. Before experiments, animals were randomly allocated into control, positive control and experimental groups (five animals per group, *n* = 5). Before 3–4 h of tests, access to food was restricted for the experimental animals but free access to water was ensured. Appropriate steps were taken to diminish their sufferings.

### Treatments

The animals of control group received 0.5% Na-CMC by oral administration at the dose of 10 mL/Kg b.w., 30 min before experiments. For the positive control group mice, standard drug, morphine (5 mg/Kg b.w.) or diclofenac sodium (10 mg/Kg b.w.) was intraperitoneally (i.p.) administered 15 min before experiments. The experimental group mice received oral dose (50, 100 or 200 mg/Kg b.w) of catechin or hydro-alcoholic extract of the bark of *A. lacucha* (HEBA). The doses of (+)-catechin and HEBA were selected on the basis of trial experiments and their previously reported effective doses [18, 24, 25]. Naloxone (2 mg/Kg b.w., i.p.) and glibenclamide (10 mg/Kg b.w., i.p.) was used 15 min before morphine, HEBA or (+)-catechin administration. Na-CMC (0.5%, w/v, prepared in physiological saline) was used as a vehicle to prepare all the doses of standard drugs, experimental compound or extracts.

### Acute toxicity test

The acute oral toxicity test of HEBA was carried out on six groups of mice (*n* = 5) at the dose of 50, 100, 500, 1000, 2000 and 3000 mg/Kg according to the principle of 420 – Fixed Dose Procedure of Organization for Economic Cooperation and Development (OECD). Control group mice (n = 5) received vehicle at the dose of 10 ml/Kg b.w, (p.o.). Both the experimental and control groups were provided with free access to food and water ad libitum. The mice were observed for mortality, allergic reactions, and any abnormal behaviors 14 days after treatment. On the 15th day the mice were sacrificed and their vital organs including heart, liver, lung, stomach, and kidneys were subjected to macroscopic evaluation [26].

### Antinociceptive experiments

#### Hot plate test

The central nociceptive activity of HEBA and (+)-catechin was assessed using Eddy’s hot plate apparatus (Kshitij Innovations, Haryana, India), according to the method described by Eddy and Leimbach (1953) [27]. Animals were placed individually on the surface of the heated metal plate which was maintained at 55 ± 0.5 °C. Responses of the mice including forepaw licking, jumping, and withdrawal of paw(s) were considered as nociception. A pre-treatment latency of each mouse on the hot plate was recorded as a baseline. The latency times of experimental mice were recorded at 30, 45, 60, 90- and 120-min following treatments with vehicle, morphine, HEBA or (+)-catechin. Mice were kept on the hot plate for a maximum time period of 20 s to avoid any tissue injuries. The percentage of maximal possible analgesic effect (MPE) was calculated using the following formula: % MPE = [(post − treatment latency – pre − treatment latency)/(cut − off time – pre − treatment latency)] × 100.

#### Tail immersion test

Tail immersion test was performed according to the method previously described by D’Amour and Smith (1941) [28]. This experiment involved immersing 1–2 cm of the tail of each mouse into hot water bath maintained at 52 ± 1 °C. The mice were immobilized using ‘Chux’ for a few seconds. The latency time of tail withdrawal from hot water was measured and was used as an index of nociception. The tail of the mice was immersed for a maximum time period of 20 s to avoid tissue injuries. A latency was recorded before administration of vehicle, morphine, HEBA or (+)-catechin. The nociceptive reactions were recorded at 30, 45, 60, 90- and 120-min following treatments. The % MPE was measured using the formula as mentioned in the hot plate test.

#### Acetic acid-induced writhing test

The test was conducted according to the procedure described by Rauf et al. (2016) [29] with slight modification. Briefly, the writhing was induced by administrating 1% (w/v) acetic acid (10 ml/Kg, b.w., i.p.) after 30 min of vehicle, HEBA, (+)-catechin or 15 min of diclofenac sodium (10 mg/Kg, i.p.) administration. The individual mice were observed for nociceptive responses including contraction of the abdominal muscle and stretching of the hind limbs. The number of writhings were counted during a 30 min time period following the first response. The percentage inhibition of writhing was calculated using the following formula: % Inhibition = [{Mean no. of writhes (Control) − Mean no. of writhes (Test)}/{Mean no. of writhes (Control)}] × 100.

#### Formalin-induced nociception test

A formalin solution (5% formalin in 0.9% saline, 25 μL) was injected subcutaneously into the plantar surface of the right hind paw of each mouse to induce pain after 15 min of morphine and 30 min of vehicle, HEBA, (+)-catechin treatments. The licking, biting responses of the injected paw were considered as nociception. The time of responses were measured every 5 min, for a period of 45 min. Edema (Δ) of the injected paw was calculated from its thickness which was measured before and 60 min after the formalin administration using a digital slide caliper. The inhibition of the degree of edema was estimated according to following equation: Δ = (paw thickness after formalin injection – paw thickness before formalin injection) [30].

#### Carrageenan-induced paw edema test

The test was performed according to the procedure described by Winter et al. (1962) [31]. One hundred microliters of 1% (w/v) suspension of λ-carrageenan was administered into the sub-plantar region of the right hind paw of each mouse after diclofenac sodium, vehicle, HEBA or (+)-catechin administration. The paw thickness (mm) of each mouse was measured using digital slide caliper before (C_b_) and at 0, 1, 2, 3, 4, 5, 6 h., after (C_a_) carrageenan administration to determine the degree of edema (Δ). The percent inhibition of edema in was determined by using following formula: % Inhibition = [{(C_a_ − C_b_)_control_ – (C_a_ − C_b_)_test_}/(C_a_ − C_b_)_control_}] × 100.

### Analysis of possible mechanism of action

#### Involvement of opioid receptors

The possible participation of opioid receptors in the antinociceptive effect of HEBA and (+)-catechin was investigated according the procedure described by Khan et al., (2011) [32]. Briefly, naloxone (2 mg/Kg, b.w., i.p.), a non-selective opioid receptor antagonist, was administered, 15 min before the treatments of HEBA or (+)-catechin in tail immersion and hot plate test. The latency times were recorded as discussed in the previous sections.

#### Involvement of ATP-sensitive K^+^ channel system

The possible involvement of ATP-sensitive K^+^ channel system in the antinociceptive effect of HEBA and (+)-catechin was determined according to the procedure described by Perimal et al. (2011) [33]. Mice received glibenclamide (10 mg/Kg, i.p.), an ATP-sensitive K+ channel blocker, 15 min before the treatments of HEBA or (+)-catechin. After 30 min of treatments, they were treated with 1% (w/v) acetic acid. The writhing response was counted as discussed in the acetic acid-induced writhing test.

### Statistical analysis

Results are presented as mean ± SEM. Statistical analysis was performed by one-way or two-way analysis of variance (ANOVA) followed by Dunnett’s or Bonferroni’s test as appropriate post hoc test, using SPSS 22 software (IBM, USA). *p* < 0.05 was considered as statistically significant.

## Results

### Phytochemical analysis

Preliminary phytochemical analysis of HEBA revealed the presence of flavonoids, carbohydrates, glycosides, saponins, tannins, and proteins. The total flavonoid and phenolic contents of the plant extract were found to be 363.34 ± 0.63 mg (equivalent to quercetin) and 292.06 ± 6.07 mg (equivalent to gallic acid) per gm extract, from the regression equation y = 0.014x + 0.3999, R^2^ = 0.9978 and y = 0.0063x + 0.052, R^2^ = 0.9982, respectively.

### Acute toxicity

In oral acute toxicity test, mortality, allergic reaction or behavioral changes were nor seen in the animals for 14 days observation period after the administration of HEBA at 500–3000 mg/Kg b.w. dose. There were no signs of injury or adverse effects in the organs from macroscopic evaluation. Therefore, it can be suggested that HEBA possess low toxicity profile.

### Hot plate

**Fig. 2:**
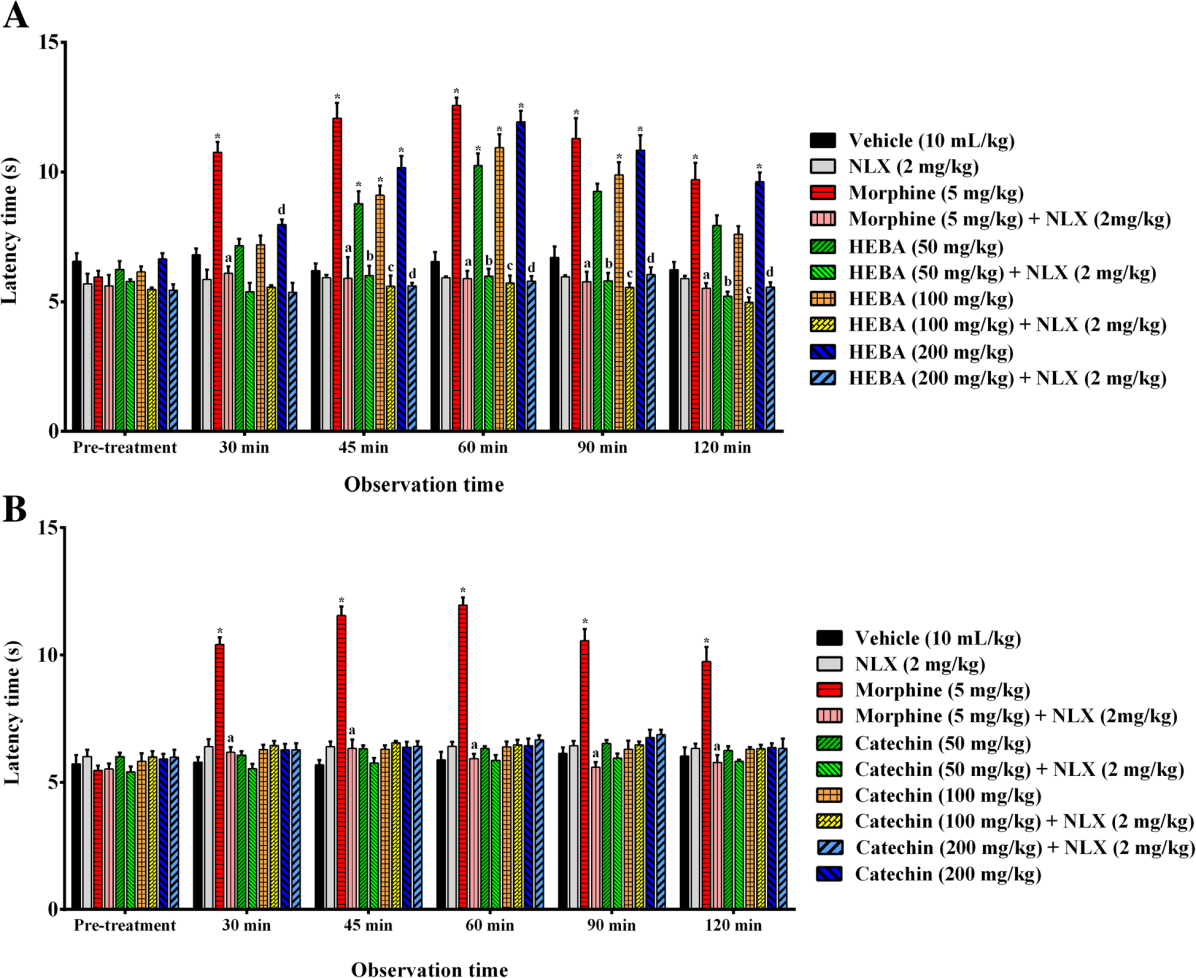
Effect of morphine, HEBA, (+)-catechin and naloxone treatments on latent time in hot plate test. Panel **a** shows the effect of HEBA and naloxone pre-treatment on latency. Panel **b** shows the effect of (+)-catechin and naloxone pre-treatment on latent time. Data are presented as mean ± SEM (*n* = 5). ^*^ represents *p* < 0.001, compared to control group mice. a, b, c and d represent *p* < 0.001, compared to 50, 100 and 200 mg/Kg b.w. dose treatment group respectively. HEBA = hydro-methanolic extract of the bark of *A. lacucha*. NLX = naloxone

Oral treatment of HEBA (Fig. 2a) significantly (*p* < 0.001) increased the latency time at all experimental doses with respect to vehicle-treated animals in hot plate test. On the other hand, (+)-catechin treatment did not induce any significant change in latency time (Fig. 2b). HEBA demonstrated dose dependent effect which was highest at the dose of 200 mg/Kg b.w. at all observational periods. However, standard drug, morphine exerted the highest effect throughout the experimental session. Morphine demonstrated maximum latency (12.57 ± 0.30s, 47.12 ± 2.29% inhibition, Fig. 2a; 11.95 ± 0.36 s, 44.55 ± 2.75% inhibition, Fig. 2b) at 60 min. The plant extract and catechin, induced maximum latency 11.93 ± 0.43 s (39.52 ± 3.30% inhibition) and 6.75 ± 0.31 s (6.24 ± 2.37% inhibition) at 60 and 90 min, respectively at the dose of 200 mg/Kg b.w. Naloxone did not cause a substantial change in the latency time of (+)-catechin (Fig. 2b) compared to control group but significantly (*p* < 0.001) inhibited the effect of morphine and HEBA (Fig. 2a).

### Tail immersion

**Fig. 3:**
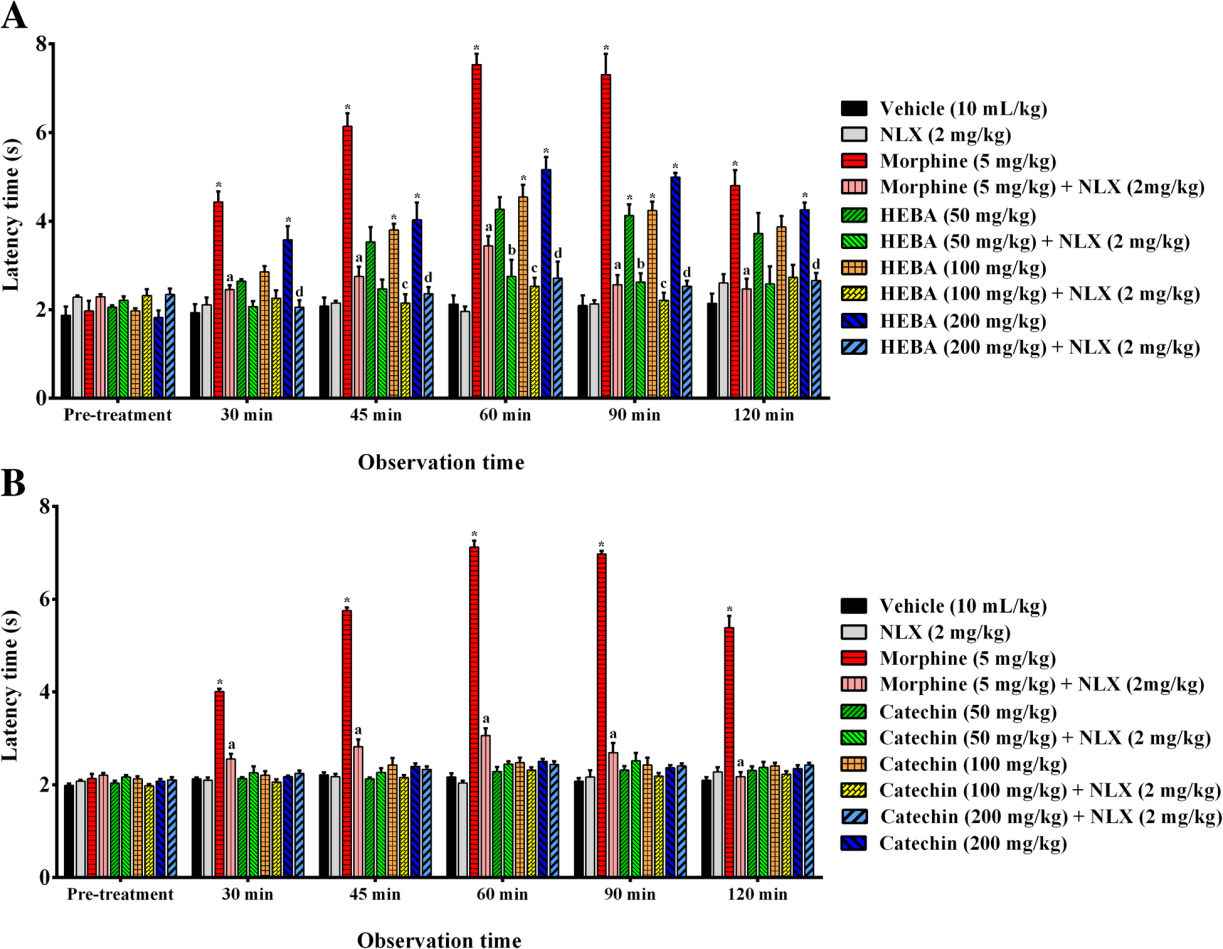
Effect of morphine, HEBA, (+)-catechin and naloxone treatments on latent time in tail immersion test. Panel **a** shows the effect of HEBA and naloxone pre-treatment on latency. Panel **b** shows the effect of (+)-catechin and naloxone pre-treatment on latent time. Data are presented as mean ± SEM (*n* = 5). ^*^ represents *p* < 0.001, compared to control group mice. a, b, c and d represent *p* < 0.001, compared to 50, 100 and 200 mg/Kg b.w. dose treatment group respectively. HEBA = hydro-methanolic extract of the bark of *A. lacucha*. NLX = naloxone

HEBA (Fig. 3a) administration (p.o.) caused significant (*p* < 0.001) increase in thermal latency, compared to control group mice in a dose-dependent manner. (+)-Catechin treatment did not produce a significant change in thermal latency at any experimental doses. The greatest effect against the thermal nociception was observed on 60 min by HEBA (5.16 ± 0.29 s latency, 18.30 ± 2.27% inhibition), and catechin (2.51 ± 0.05 s latency, 2.41 ± 0.32% inhibition) at the dose of 200 mg/Kg b.w. HEBA (200 mg/Kg, b.w.) demonstrated significant (*p* < 0.001) increase of thermal latency time as well as inhibition of nociception at all observational periods. However, morphine administration had a significant (*p* < 0.001) effect throughout the experimental session and was highest at 60 min which happened to be higher than any treatments of HEBA or (+)-catechin as shown in Fig. 3a (7.53 ± 0.25 s latency, 30.83 ± 0.53% inhibition) and 3B (7.12 ± 0.14 s latency, 27.92 ± 0.68% inhibition). Naloxone caused a significant (*p* < 0.01) reduction in thermal latency as well as % inhibition of nociception of morphine, HEBA (Fig. 3a) without altering the thermal nociceptive effect by itself or (+)-catechin (Fig. 3b).

### Acetic acid-induced writhing

**Fig. 4:**
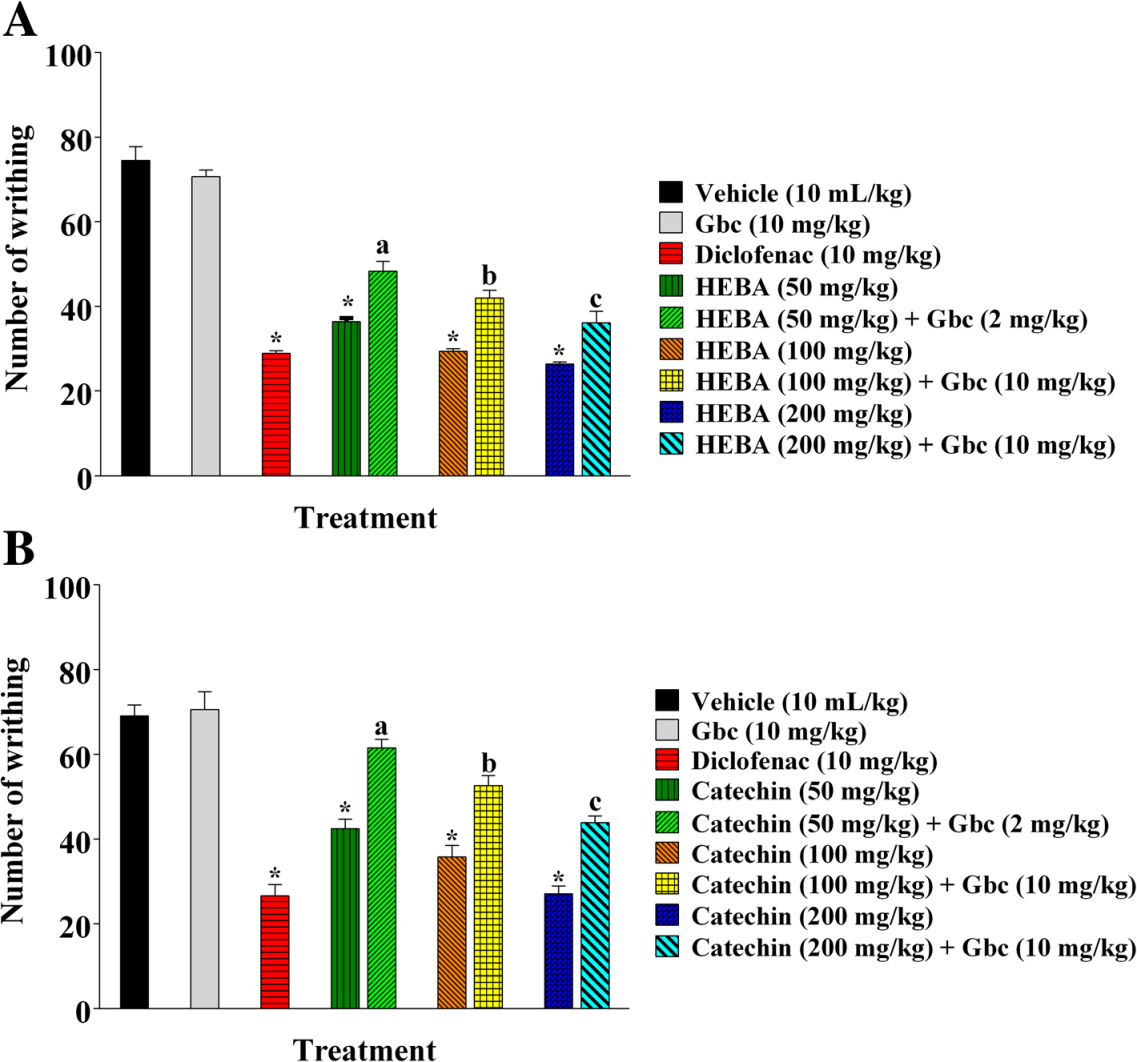
Effect of diclofenac, HEBA, (+)-catechin and glibenclamide treatments in the acetic acid-induced writhing test. Panel **a** shows the effect of HEBA and glibenclamide pre-treatment on writhing. Panel **b** shows the effect of (+)-catechin and glibenclamide pre-treatment on writhing. Data are presented as mean ± SEM (*n* = 5). ^*^ represents *p* < 0.001, compared to control group mice. a, b, and c represent *p* < 0.01, compared to 50, 100 and 200 mg/Kg b.w. dose treatment group respectively. HEBA = hydro-methanolic extract of the bark of *A. lacucha*. Gbc = glibenclamide

As illustrated in Fig. 4, diclofenac, HEBA (Fig. 4a) and (+)-catechin (Fig. 4b) treatment (p.o.) showed significant (*p* < 0.001) reduction of writhing episodes at all experimental doses in a dose-dependent manner, compared to control group mice. The plant extract and catechin treatment showed the highest reduction of writhing episodes (26.4 ± 0.48, 64.56% inhibition and 27.1 ± 1.81, 60.78% inhibition), at the dose of 200 mg/Kg b.w. which were closer to diclofenac treatment group (28.9 ± 0.62, 64.56% inhibition and 26.6 ± 2.68, 61.51% inhibition) as depicted in Fig. 4a and b, respectively.

#### Involvement of ATP-sensitive K^+^ channel system in antinociceptive activity

Glibenclamide treatment significantly (*p* < 0.01) reversed the writhing inhibition effect of HEBA (Fig. 4a) and catechin (Fig. 4b). However, glibenclamide could not cause significant alteration of the number of writhing (70.70 ± 1.52 and 70.60 ± 4.15), compared to control group (74.50 ± 3.28 and 69.1 ± 2.54) as shown in Fig. 4a and b, respectively.

### Formalin-induced nociception

**Fig. 5:**
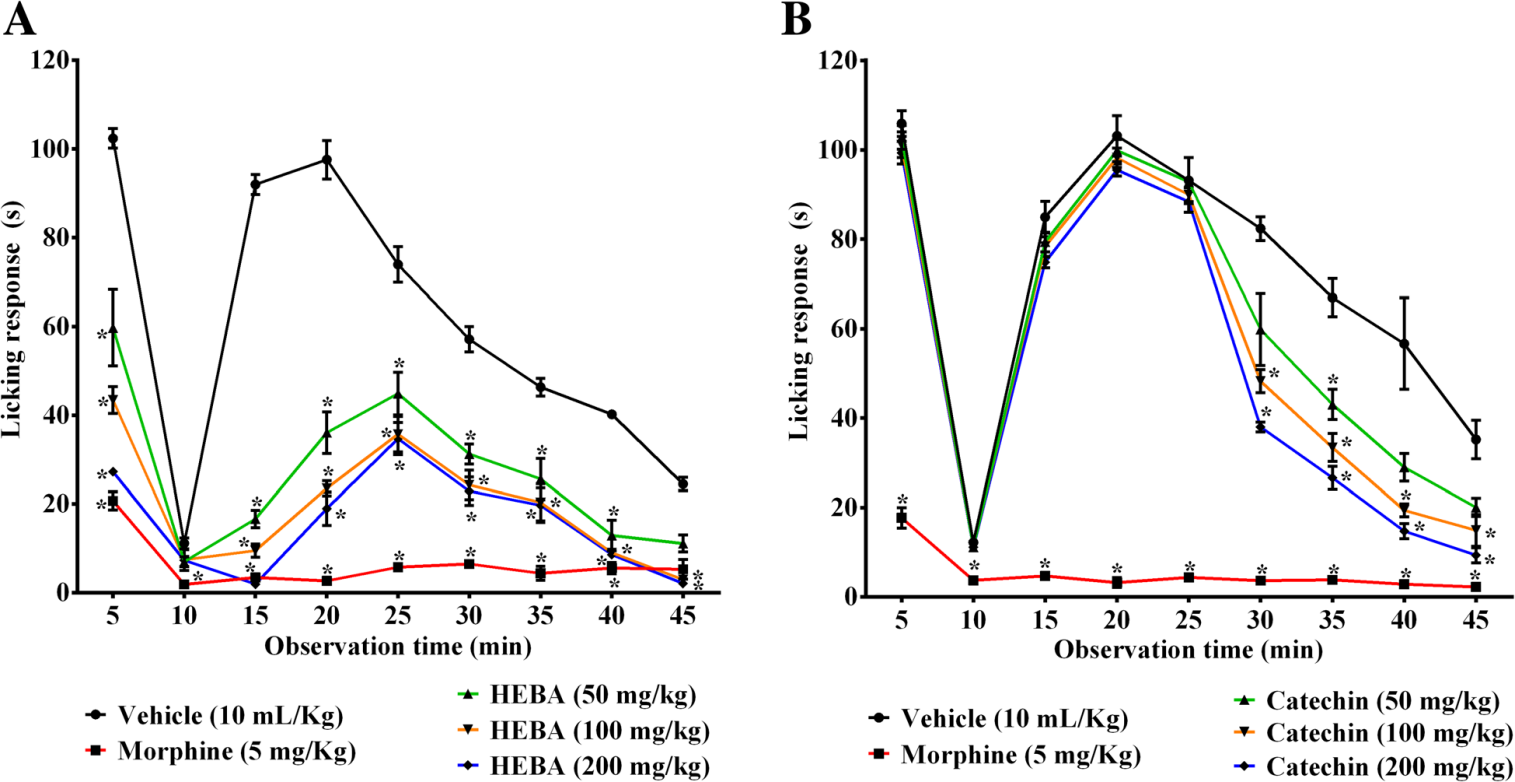
Effect of morphine, HEBA (panel **a**) and (+)-catechin (panel **b**) treatments on licking time at multiple time interval in formalin-induced nociception test. Data are presented as mean ± SEM (*n* = 5). ^*^ represents *p* < 0.001 at the same time interval, compared to control group mice. HEBA = hydro-methanolic extract of the bark of *A. lacucha*

Oral ingestion of HEBA and (+)-catechin caused significant (*p* < 0.001) inhibition of formalin-induced licking and edematogenic responses, compared to vehicle-treated mice as shown in Tables 1 and 2, respectively. However, significant protection of the nociception was not found by the (+)-catechin treatments in early phase. The inhibition of late phase (11–40 min) nociceptive response by HEBA and catechin was more pronounced than that of the early phase (0–10 min) and was maximum at the dose of 200 mg/Kg, b.w. Morphine treatment demonstrated significant (*p* < 0.001) reduction of nociception in both phases and edematogenic response, compared to control group (Tables 1 and 2). A two-way ANOVA analysis revealed that there was a significant effect of time (F = 102.186, *p* < 0.001), group (F = 385.641, *p* < 0.001) and time × group interaction (F = 17.617, *p* < 0.001) for HEBA treatment. There was also significant effect of time (F = 312.163, *p* < 0.001), group (F = 434.370, *p* < 0.001) and time × group interaction (F = 20.155, *p* < 0.001)) for (+)-catechin treatment. Post hoc analysis at each time point demonstrated that the reduction of licking responses by HEBA (100 and 200 mg/Kg b.w.) was dose dependent as well as significant (*p* < 0.001) over the experimental session (Fig. 5)a. (+)-Catechin treatment could induce significant (*p* < 0.001) reduction of nociception after 30 min of formalin treatment at the doses of 100 and 200 mg/Kg b.w. (Fig. 5b).

**Table 1:** Effect of hydro-alcoholic extract of *A. lacucha* (HEBA) in formalin-induced nociception test

Treatment	Dose (mg/Kg)	Nociceptive response (s)				Edema thickness (mm)	% inhibition
		Early Phase (0–10 min)	% inhibition	Late Phase (11–45 min)	% inhibition		
Vehicle	–	113.56 ± 1.82	–	407.21 ± 5.46	–	1.45 ± 0.07	–
Morphine	5	22.59 ± 2.27^*^	80.11	28.33 ± 2.43^*^	93.04	0.78 ± 0.06^**^	45.93
HEBA	50	66.73 ± 9.43^*^	41.24	167.34 ± 9.61^*^	58.91	1.10 ± 0.03^*^	24.00
HEBA	100	50.87 ± 1.96^*^	55.21	122.45 ± 8.78^*^	69.93	0.94 ± 0.06^*^	35.31
HEBA	200	34.59 ± 0.65^*^	69.54	106.80 ± 4.05^*^	73.77	0.85 ± 0.07^*^	41.10

Data are presented as mean ± SEM (*n* = 5). HEBA = hydro-methanolic extract of *A. lacucha* bark. ^*^ and ^**^ represents *p* < 0.001 and *p* < 0.01 compared to control group mice

**Table 2:** Effect of catechin and morphine in formalin-induced nociception test

Treatment	Dose (mg/Kg)	Nociceptive response (s)				Edema thickness (mm)	% inhibition
		Early Phase (0–10 min)	% inhibition	Late Phase (11–45 min)	% inhibition		
Vehicle	–	118.18 ± 2.80	–	487.37 ± 17.41	–	1.37 ± 0.03	–
Morphine	5	21.45 ± 2.29^*^	81.35	22.65 ± 1.37*	95.35	0.72 ± 0.04^*^	47.67
(+)-Catechin	50	114.05 ± 2.62	3.49	404.17 ± 13.08	17.07	1.34 ± 0.01	2.62
(+)-Catechin	100	112.43 ± 2.28	4.86	377.18 ± 7.11*	22.61	1.11 ± 0.02^*^	19.24
(+)-Catechin	200	110.56 ± 2.38	6.45	338.19 ± 3.48*	30.61	0.92 ± 0.01^*^	33.24

Data are presented as mean ± SEM (n = 5). ^*^ represents *p* < 0.001, compared to control group mice

### Carrageenan-induced paw edema

**Fig. 6:**
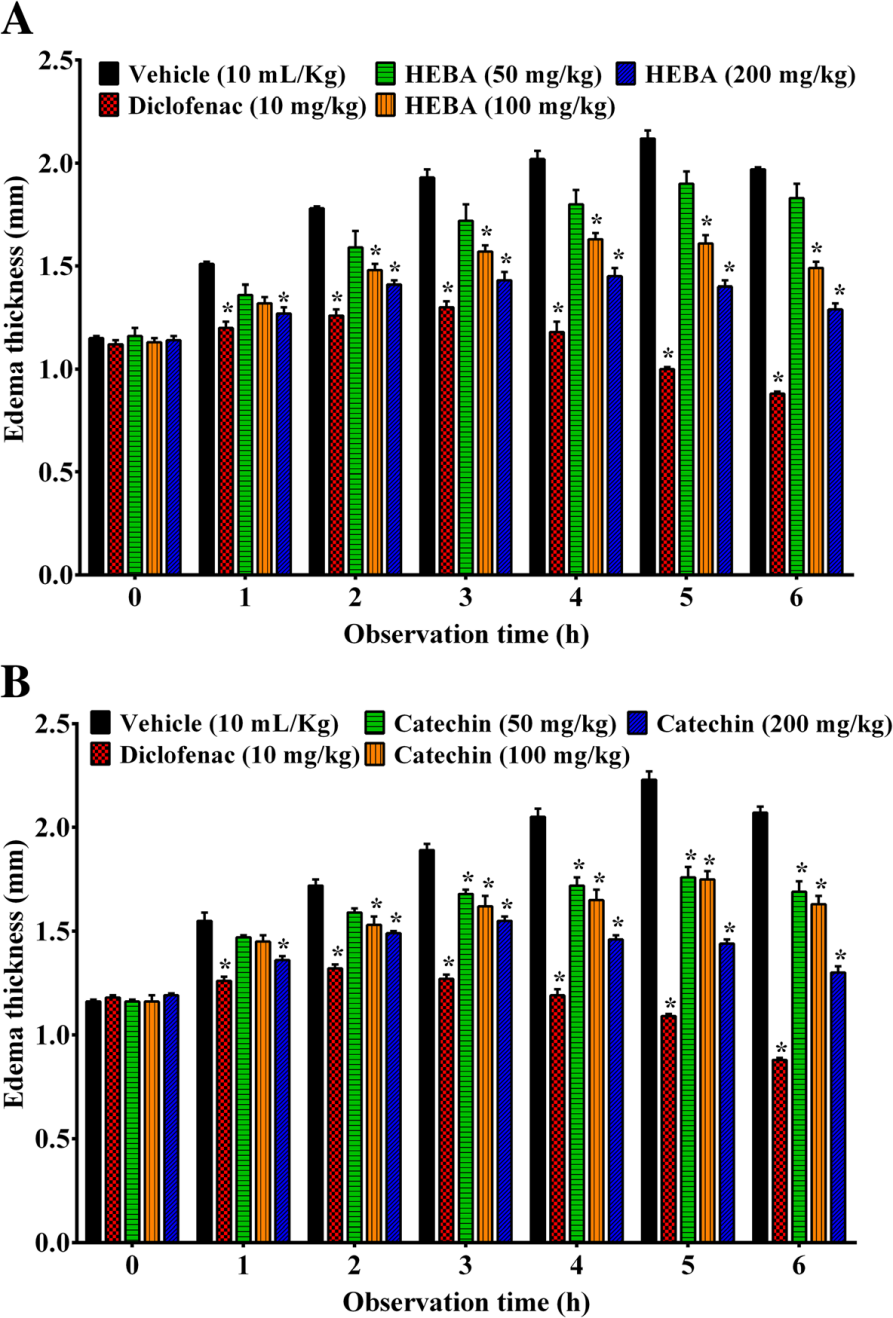
Effect of diclofenac, HEBA (panel **a**) and (+)-catechin (panel **b**) treatments on paw edema at multiple time interval in carrageenan-induced paw edema test. Data are presented as mean ± SEM (*n* = 5). ^*^ represents *p* < 0.001, compared to control group mice. HEBA = hydro-methanolic extract of the bark of *A. lacucha*

As demonstrated in Fig. 6, carrageenan treatment caused a gradual increase of edematogenic response which peaked at the 5th h (2.12 ± 0.04 mm, Fig. 6a; .23 ± 0.04 mm, Fig. 6b) and declined at 6th h (1.97 ± 0.01 mm, Fig. 6a; 2.07 ± 0.03, Fig. 6b). Oral ingestion of HEBA (Fig. 6a) and (+)-catechin (Fig. 6b) significantly (*p* < 0.001) diminished the carrageenan-induced paw edema from 1st h to end of the experimental session at the dose of 200 mg/Kg with respect to the control group. (+)-Catechin also caused significant (*p* < 0.001) inhibition at 50 mg/Kg b.w. dose from 3rd h to end of the experimental session. Both the plant extracts and (+)-catechin showed dose-dependent inhibition and the inhibitory effect was highest at 6 h with (1.29 ± 0.03 mm) 34.55 and (1.30 ± 0.03 mm) 37.07%, respectively. Diclofenac treatment demonstrated significant (*p* < 0.001) inhibition from 1st h to end of the experimental session and the effect was highest at 6 h with 55.32 (Fig. 6a) and 57.72% (Fig. 6b) inhibition which was greater than both of HEBA and (+)-catechin treatments.

## Discussion

The present study analyzed antinociceptive effect of *A. lacucha* bark and its isolated compound in different chemical and thermal pain models in order to justify its potential pain protection effect as described in traditional system of medicine. As previously reported, aqueous and methanol extract of the plant extract possess phenolics among which some of them have promising in-vitro anti-inflammatory activity. Therefore, bark of the plant has been subjected to hydro-methanolic extraction, which has been found to contain substantial amount of phenolics as well as flavonoids. Furthermore, isolation of active compound, (+)-catechin (characterized by high resolution 1D and 2D NMR spectroscopy), from the hydro-methanolic extract of *A. lacucha* bark (HEBA) by successive applications of different chromatographic techniques including VLC followed by PTLC was done. The HEBA demonstrated significant antinociceptive effect at the doses of 50–200 mg/Kg b.w. which have been found to be non-toxic in experimental animals.

The hot-plate and tail immersion tests are the most widely used models for evaluating the centrally acting analgesic activity. Both tests detects the analgesic responses mediated by different mechanisms even though thermal stimulus is used as a source of nociception in both models [34]. The thermal stimulus evokes spinally mediated nociception in tail immersion test whereas the hot plate test induces supraspinally mediated nociception [35]. The action of opioid drugs has been reported to involve spinal (δ_1,_ μ_1_, κ_3_) as well as supraspinal (σ_2_, μ_1_, δ_1_, κ_3_) receptors [36–38]. In the present study, the treatment with HEBA caused a significant increase in thermal latency in both hot plate (Fig. 2a) and tail immersion test (Fig. 3a). The outcome of the results suggests that the inhibition of thermal nociception by HEBA could be mediated via spinal and supraspinal systems. However, the effect was absent for the isolate of the plant extract, (+)-catechin, treatments in both experiments (Figs. 2b and 3b). The non-selective opioid receptor antagonist, naloxone, inhibited the effect of the plant extract in both tests which further confirms the involvement of opioid receptors in its antinociceptive effect.

The acetic acid-induced writhing test is a visceral pain model, which is frequently used to evaluate analgesic effects of new drug candidates [39]. Acetic acid administration leads to the direct activation of non-selective cation channels, indirect release of endogenous nociceptive mediators as well as PGs, histamine, bradykinin, serotonin, eicosanoids, cytokines in the peritoneal fluid and stimulates peripheral nociceptive neurons which results in a pain syndrome characterized by writhing [40, 41]. Inflammatory pain sensation in acetic acid-induced abdominal constriction response is also due to the release of arachidonic acid metabolites from tissue phospholipids upon the action of COX enzyme, and prostaglandin (PGE_2_ and PGF_2_) biosynthesis [42]. The results reported herein indicated that oral administration of HEBA (Fig. 4a) and (+)-catechin (Fig. 4b) significantly (*p* < 0.001) reduced the number of abdominal constriction induced by acetic acid. Therefore, antinociceptive effect of HEBA and (+)-catechin could be due to the inhibition of the synthesis of arachidonic acid metabolites, visceral inflammatory mediators and/or suppression of peripheral nociceptive neurons. The cyclooxygenases (COX-1 and COX-2) inhibitory effect of catechin, described by the previous report, support the results of the present investigation. It is also evident from the result that, the ATP-sensitive K^+^ channel blocker, glibenclamide significantly (*p* < 0.01) attenuated the writhing inhibitory action of both HEBA (Fig. 4a) and catechin (Fig. 4b). Glibenclamide blocks the ATP-sensitive K^+^ channel without interrupting the voltage-gated and Ca^2+^ actuated K^+^ channels [43, 44]. Thus, it can be suggested that antinociceptive effect of HEBA and (+)-catechin involves opening of the ATP-sensitive K^+^ channel followed by an efflux of K^+^ ions and suppression of excitability of cellular membrane by hyperpolarization and/or repolarization [45].

The formalin injection produces a licking response, regarded as nociception, generally mediated by two distinct phases. The first phase is characterized as early or neurogenic phase, which begins instantly after formalin injection and the second phase as inflammatory phase, which starts 10 min after the formalin injection. Neurogenic phase involves centrally mediated pain, which is associated with the direct excitation of nociceptive afferent fibers, predominantly C fibers. The inflammatory phase nociception is induced by the release of different mediators such as histamine, PGs, serotonin and bradykinin the peripheral tissues and functional changes in the spinal dorsal horn [46, 47]. The release of bradykinin stimulates the nociceptive afferent nerve terminals which are responsible for the development of plasma extravasation as well as paw edema formation [48]. In the present study, HEBA (Table 1) and (+)-catechin (Table 2) significantly (*p* < 0.001) reduced the formalin-induced paw swelling as well as licking response. Inhibition rate of the licking response was found to be more prominent in the second phase. Therefore, the effect of HEBA and catechin could be attributed to the inhibition of inflammatory mediators release in the peripheral tissues. The results indicated that the plant extract treatments significantly (*p* < 0.001) diminished the nociceptive effect at both phases (Table 1) where (+)-catechin treatments showed significant action only at late phase (Table 2). Previous reports found that centrally acting analgesics (opioids) protects the nociception of both neurogenic and inflammatory phases whereas peripherally acting analgesics (e.g. indomethacin, acetylsalicylic acid) mainly protects the nociception of inflammatory phase [46, 49]. Therefore, the result suggests that HEBA possess central and peripheral antinociceptive effects whereas the (+)-catechin is able to diminish peripheral nociception. These outcomes also support the aforementioned results of tail immersion, hot plate, and acetic-acid-induced writhing tests.

Carrageenan injection liberates a variety of chemical mediators and is characterized as a biphasic acute inflammatory response [50]. The edema of initial phase (0–1 h) is associated with the release of histamine, serotonin, and bradykinin. The late phase occurs after the first hour of carrageenan injection and is continued for 5 h, which correlates with the increased edema formation and elevated production of TNF-α, NO, and PGs [51]. In addition, the late phase has been reported to induce cyclooxygenase-2 (COX-2) production [52]. The plant extract (Fig. 6a) and (+)-catechin (Fig. 6b) significantly (*p* < 0.001) restrained the edematous response in both the initial phase and late phase of inflammation. The result indicates that the effect of the plant extract and (+)-catechin could be due to the inhibition of liberation of histamine, serotonin, and bradykinin. In addition, their edema inhibitory effect could be also associated with the inhibition of synthesis of COX-2 as well as cytokine, TNF-α, and NO.

## Conclusions

The results of the present investigation showed that the hydro-alcoholic extract of the bark of *A. lacucha* possesses promising antinociceptive activity, which complies with its application in the traditional system of medicine. Its isolated polyphenolic-flavonoid, (+)-catechin, also exhibited significant protection from pain, which could be partially responsible for the effect demonstrated by the extract. The possible association of opioid receptors in the pain protection effect of the plant extract was confirmed from the antagonist effect of naloxone. Both the plant extract and its isolate (+)-catechin exerted antinociceptive action via ATP-sensitive K^+^ channel. The results also indicate that the action of the plant extract and its isolate is due to the inhibition of inflammatory mediators including, cyclooxygenases (COX), PGs, histamine, bradykinin, and serotonin. In the present study, it has been noticed that the plant extract has exerted opioid-mediated analgesic effect whereas such effect was absent for its isolated compound (+)-catechin. Therefore, isolation of further bioactive compounds, as well as investigations regarding their direct modulation of the receptor, will be required to provide clear insight of the mechanism of the action of the plant. The present findings demonstrated that the bark of *A. lacucha* and its isolate, (+)-catechin could be potential candidates for the further research regarding the development of analgesic therapeutics.

## Additional file

Additional file 1: Antinociceptive activities of *Artocarpus lacucha* Buch-Ham (Moraceae) and its isolated phenolic compound, catechin, in mice. **Figure S1.** Structure of (+)-catechin. **Table S1.** ^1^H NMR, ^13^C NMR, COSY, HSQC and HMBC data of (+)-catechin. **Figure S2.** ^1^H NMR (400 MHz, CD_3_OD) spectrum of (+)-catechin. **Figure S3.** ^13^C NMR (100 MHz, CD_3_OD) spectrum of (+)-catechin. **Figure S4.** DEPT NMR (100 MHz, CD_3_OD) spectrum of (+)-catechin. **Figure S5.** COSY NMR (400 MHz, CD_3_OD) spectrum of (+)-catechin. **Figure S6.** HSQC NMR spectrum of (+)-catechin. **Figure S7.** HMBC NMR spectrum of (+)-catechin. (DOCX 2993 kb)

## Abbreviations

ANOVA: One-way analysis of variance
ATP: Adenosine triphosphate
AVMA: American Veterinary Medical Association
COSY: Correlation spectroscopy
COX: Cyclooxygenase
DEPT: Distortion less Enhancement by Polarization Transfer
HEBA: Hydro-methanolic extract of bark of *Artocarpus lacucha*
HMBC: Heteronuclear multi-bond connectivity
HSQC: Heteronuclear single quantam coherence
i.p.: intraperitoneal
icddr,b: International Center for Diarrhoeal Disease Research, Bangladesh
MPE: Maximal possible analgesic effect
NMR: Nuclear magnetic resonances
NO: Nitric oxide
OECD: Organization for Economic Cooperation and Development
p.o.: per oral
PGs: Prostaglandins
PTLC: preparative thin layer chromatography
R_f_: Retention factor
SEM: Standard error of mean
TLC: Thin layer chromatography
TNF-α: tumor necrosis factor-alpha
UV: Ultra violet
VLC: Vacuum liquid chromatography

## References

[1] Martínez-González CL, Martínez L, Martínez-Ortiz EJ, González-Trujano ME, Déciga-Campos M, Ventura-Martínez R (2017). Moringa oleifera , a species with potential analgesic and anti-inflammatory activities. Biomed Pharmacother.

[2] Watkins LR, Milligan ED, Maier SF (2003). Glial proinflammatory cytokines mediate exaggerated pain states: implications for clinical pain. Adv Exp Med Biol.

[3] Oliveira TLS, de MSR, de SS, de OMG, Florentino IF, da SDM (2017). Antinociceptive, anti-inflammatory and anxiolytic-like effects of the ethanolic extract, fractions and Hibalactone isolated from Hydrocotyle umbellata L. (Acariçoba) – Araliaceae. Biomed Pharmacother.

[4] Tiwari A, Singh A (2014). Synthesis and antinociceptive activity of novel mannich base derivatives of some new fused 3,5-pyrazolidinedione. J Adv Pharm Technol Res.

[5] Poetker David M., Reh Douglas D. (2010). A Comprehensive Review of the Adverse Effects of Systemic Corticosteroids. Otolaryngologic Clinics of North America.

[6] Kifayatullah M, Mustafa MS, Sengupta P, Sarker MMR, Das A, Das SK (2015). Evaluation of the acute and sub-acute toxicity of the ethanolic extract of Pericampylus glaucus (lam.) Merr. In BALB/c mice. J Acute Dis.

[7] Rauf A, Jehan N, Ahmad Z, Mubarak MS. Analgesic potential of extracts and derived natural products from medicinal plants. Pain Reli - From Analg to Altern Ther Rijeka: InTech. 2017:339–51.

[8] Jagtap U.B., Bapat V.A. (2010). Artocarpus: A review of its traditional uses, phytochemistry and pharmacology. Journal of Ethnopharmacology.

[9] Ghani A (2003). Medicinal plants of Bangladesh: chemical constituents and uses. 2nd edn. Med. Plants Bangladesh Chem. Const. Uses. Dhaka: Asiatic Society of Bangladesh.

[10] Uprety Y, Poudel RC, Asselin H, Boon E (2011). Plant biodiversity and ethnobotany inside the projected impact area of the upper Seti hydropower project, Western Nepal. Environ Dev Sustain.

[11] Janick J, Paull RE. The encyclopedia of fruit and nuts. Choice Rev Online Wallingford: CABI. 2008.

[12] Ali RM, Samah ZA, Mustapha NM, Hussein N. ASEAN herbal and medicinal plants. Jakarta: ASEAN Secretariat. 2010.

[13] Gautam P, Patel R (2014). Artocarpus lakoocha Roxb: an overview. Eur J Complement Altern Med.

[14] Pandey A, Bhatnagar SP (2009). Antioxidant and phenolic content of the bark of Artocarpus Lakoocha. Pharma Rev.

[15] Su BN, Cuendet M, Hawthorne ME, Kardono LBS, Riswan S, Fong HHS (2002). Constituents of the bark and twigs of Artocarpus dadah with cyclooxygenase inhibitory activity. J Nat Prod.

[16] Saowakon N, Tansatit T, Wanichanon C, Chanakul W, Reutrakul V, Sobhon P (2009). Fasciola gigantica: anthelmintic effect of the aqueous extract of Artocarpus lakoocha. Exp Parasitol.

[17] Kirtikar K, Basu B. Indian medicinal plants. 2nd edn. Indian med. Plants. Uttaranchal: oriental enterprises; 2007.

[18] Tang L-Q, Wei W, Wang X-Y (2007). Effects and mechanisms of catechin for adjuvant arthritis in rats. Adv Ther.

[19] Du X, Wang C, Zhang H (2011). Activation of ATP-sensitive potassium channels antagonize nociceptive behavior and hyperexcitability of DRG neurons from rats. Mol Pain SAGE Publications.

[20] Selim SA, Adam ME, Hassan SM, Albalawi AR (2014). Chemical composition, antimicrobial and antibiofilm activity of the essential oil and methanol extract of the Mediterranean cypress (Cupressus sempervirens L.). BMC Complement Altern Med.

[21] Singleton VL, Orthofer R, Lamuela-Raventós RM (1999). Analysis of total phenols and other oxidation substrates and antioxidants by means of folin-ciocalteu reagent. Methods Enzymol.

[22] Hirose Y, Yamaoka H, Nakayama M (1990). Oxidation product of (+)-catechin from lipid peroxidation. Agric Biol Chem Taylor & Francis.

[23] Kashiwada Y, Toshika K, Chen R, Nonaka G, Nishioka I (1990). Tannins and related compounds. XCIII. Occurrence of enantiomeric proanthocyanidins in the Leguminosae plants, Cassia fistula L. and Cassia Javanica L. Chem Pharm Bull The Pharmaceutical Society of Japan.

[24] Nesa ML, Munira S, Bristy AS, Islam M, Chayan H, Rashid M (2015). Cytotoxic, anti-inflammatory, analgesic, CNS depressant, antidiarrhoeal activities of the methanolic extract of the Artocarpus Lakoocha leaves. World J Pharm Sci.

[25] Siegers CP, Völpel M, Scheel G, Younes M (1982). Effects of dithiocarb and (+)-catechin against carbon tetrachloride-alcohol-induced liver fibrosis. Agents Actions.

[26] Déciga-Campos M, Mata R, Rivero-Cruz I (2017). Antinociceptive pharmacological profile of Dysphania graveolens in mouse. Biomed Pharmacother.

[27] Eddy NB, Leimbach D (1953). Synthetic analgesics. II. Dithienylbutenyl- and dithienylbutylamines. J Pharmacol Exp Ther.

[28] D’Amour FE, Smith DL (1941). A method for determinig loss of pain sensation. J Pharmacol Exp Ther.

[29] Rauf A, Uddin G, Siddiqui BS, Khan H, Shah SUA, Ben HT (2016). Antinociceptive and anti-inflammatory activities of flavonoids isolated from Pistacia integerrima galls. Complement Ther Med.

[30] Yin Z-Y, Li L, Chu S-S, Sun Q, Ma Z-L, Gu X-P (2016). Antinociceptive effects of dehydrocorydaline in mouse models of inflammatory pain involve the opioid receptor and inflammatory cytokines. Sci Rep.

[31] Winter CA, Risley EA, Nuss GW (1962). Carrageenin-induced edema in hind paw of the rat as an assay for antiinflammatory drugs. Exp Biol Med.

[32] Khan H, Saeed M, Gilani AUH, Khan MA, Khan I, Ashraf N (2011). Antinociceptive activity of aerial parts of Polygonatum verticillatum: attenuation of both peripheral and central pain mediators. Phyther Res.

[33] Perimal EK, Akhtar MN, Mohamad AS, Khalid MH, Ming OH, Khalid S (2011). Zerumbone-induced antinociception: involvement of the l-arginine-nitric oxide-cGMP -PKC-K+ATP Channel pathways. Basic Clin Pharmacol Toxicol.

[34] Wong CH, Dey P, Yarmush J, Wu WH, Zbuzek VK (1994). Nifedipine-induced analgesia after epidural injection in rats. Anesth Analg.

[35] Chapman CR, Casey KL, Dubner R, Foley KM, Gracely RH, Reading AE (1985). Pain measurement: an overview. Pain..

[36] Hosseinzadeh H, Ramezani M, Fadishei M, Mahmoudi M (2002). Antinociceptive, anti-inflammatory and acute toxicity effects of Zhumeria majdae extracts in mice and rats. Phytomedicine..

[37] Jinsmaa Y, Fujita Y, Shiotani K, Miyazaki A, Li T, Tsuda Y (2005). Differentiation of opioid receptor preference by [Dmt1] endomorphin-2-mediated antinociception in the mouse. Eur J Pharmacol.

[38] Jinsmaa Y, Okada Y, Tsuda Y, Shiotani K, Sasaki Y, Ambo A (2004). Novel 2′,6′-dimethyl-L-tyrosine-containing pyrazinone opioid mimetic mu-agonists with potent antinociceptive activity in mice. J Pharmacol Exp Ther.

[39] Ikeda Y, Ueno A, Naraba H, Oh-Ishi S (2001). Involvement of vanilloid receptor VR1 and prostanoids in the acid-induced writhing responses of mice. Life Sci.

[40] Julius D (2001). Molecular mechanisms of nociception. Nature..

[41] Deraedt R, Jouquey S, Delevallée F, Flahaut M (1980). Release of prostaglandins E and F in an algogenic reaction and its inhibition. Eur J Pharmacol.

[42] Duarte IDG, Nakamura M, Ferreira SH (1988). Participation of the sympathetic system in acetic acid-induced writhing in mice. Brazilian J Med Biol Res.

[43] Jesse CR, Savegnago L, Nogueira CW (2007). Role of nitric oxide/cyclic GMP/K+ channel pathways in the antinociceptive effect caused by 2,3-bis(mesitylseleno)propenol. Life Sci.

[44] Lawson K (1996). Potassium channel activation: a potential therapeutic approach?. Pharmacol Ther Pergamon.

[45] Bodhinathan K, Kumar A, Foster TC (2010). Intracellular redox state alters NMDA receptor response during aging through Ca2+/calmodulin-dependent protein kinase II. J Neurosci.

[46] Hunskaar S, Hole K (1987). The formalin test in mice: dissociation between inflammatory and non-inflammatory pain. Pain..

[47] Coderre TJ, Vaccarino AL, Melzack R (1990). Central nervous system plasticity in the tonic pain response to subcutaneous formalin injection. Brain Res.

[48] Damas J, Liégeois JF (1999). The inflammatory reaction induced by formalin in the rat paw. Naunyn Schmiedeberg's Arch Pharmacol.

[49] Tjølsen A, Berge OG, Hunskaar S, Rosland JH, Hole K (1992). The formalin test: an evaluation of the method. Pain..

[50] ROSA MASSIMO (1972). Biological properties of carrageenan. Journal of Pharmacy and Pharmacology.

[51] Posadas I, Bucci M, Roviezzo F, Rossi A, Parente L, Sautebin L (2004). Carrageenan-induced mouse paw oedema is biphasic, age-weight dependent and displays differential nitric oxide cyclooxygenase-2 expression. Br J Pharmacol.

[52] Seibert K, Zhang Y, Leahy K, Hauser S, Masferrer J, Perkins W (1994). Pharmacological and biochemical demonstration of the role of cyclooxygenase 2 in inflammation and pain. Proc Natl Acad Sci U S A.

