# Supplementary material for: Antinociceptive activities of Artocarpus lacucha Buch-ham (Moraceae) and its isolated phenolic compound, catechin, in mice
Source: BMC Complement Altern Med. 2019 Aug 14;19:214. doi: 10.1186/s12906-019-2565-x (PMC6694492; doi:10.1186/s12906-019-2565-x)
Supplement: Supplementary file 1 — Antinociceptive activities of Artocarpus lacucha Buch-Ham (Moraceae) and its isolated phenolic compound, catechin, in mice. Figure S1. Structure of (+)-catechin. Table S1. 1H NMR, 13C NMR, COSY, HSQC and HMBC data of (+)-catechin. Figure S2. 1H NMR (400 MHz, CD3OD) spectrum of (+)-catechin. Figure S3. 13C NMR (100 MHz, CD3OD) spectrum of (+)-catechin. Figure S4. DEPT NMR (100 MHz, CD3OD) spectrum of (+)-catechin. Figure S5. COSY NMR (400 MHz, CD3OD) spectrum of (+)-catechin. Figure S6. HSQC NMR spectrum of (+)-catechin. Figure S7. HMBC NMR spectrum of (+)-catechin. (DOCX 2993 kb) [file 12906_2019_2565_MOESM1_ESM.docx]

**Antinociceptive activities of *Artocarpus lacucha* Buch-Ham** **(Moraceae) and its isolated phenolic compound, catechin, in mice**

**List of Tables and Figures Page no.**

**Fig. S1.** Structure of (+)-catechin……………………………………………………………… 02

**Table S1**. ^1^H NMR, ^13^C NMR, COSY, HSQC and HMBC data of (+)-catechin... 03

**Fig S2**. ^1^H NMR (400 MHz, CD_3_OD) spectrum of (+)-catechin………………… 04

**Fig S3**. ^13^C NMR (100 MHz, CD_3_OD) spectrum of (+)-catechin………………... 05

**Fig S4**. DEPT NMR (100 MHz, CD_3_OD) spectrum of (+)-catechin……………... 06

**Fig S5**. COSY NMR (400 MHz, CD_3_OD) spectrum of (+)-catechin…………….. 07

**Fig S6**. HSQC NMR spectrum of (+)-catechin…………………………………… 08

**Fig S7**. HMBC NMR spectrum of (+)-catechin…………………………………... 09

**(B)**

**(A)**

**Fig. S1.** Structure of (+)-catechin. (A) chemical structure, (B) Key HMBC correlations

**Table S1.** ^1^H NMR (400 MHz, CD_3_OD), ^13^C NMR (100 MHz, CD_3_OD), COSY, HSQC and HMBC data of (+)-catechin.

| Position no. | *δ*_H_ (mult, *J* in Hz) | *δ*_C_ | COSY | HSQC | HMBC | |
| --- | --- | --- | --- | --- | --- | --- |
|  |  |  |  |  | ^2^*J* | ^3^*J* |
| 1 | - | - | - | - | - | - |
| 2 | 4.56 (1H, d; *J* = 7.60) | 81.5 | H-3 (3.97) | C-2 (81.5) | C-3 (67.4)  C-9 (155.5)  C-1’ (130.9) | C-4 (27.1)  C-2’ (113.9)  C-6’ (118.7) |
| 3 | 3.97 (1H, ddd) | 67.4 | H-2 (4.56)  H-4α (2.86)  H-4β (2.52) | C-3 (67.4) | - | - |
| 4 (4α) | 2.86 (1H, dd) | 27.1 | H-3 (3.96)  H-4β (2.52) | 27.1 (C-4) | C-3 (67.4)  C-10 (99.5) | C-2 (81.5)  C-9 (155.5) |
| 4 (4β) | 2.52 (1H, dd) | 27.1 | H-3 (3.97)  H-4α (2.86) | 27.1 (C-4) | C-3 (67.4)  C-10 (99.5) | C-2 (81.5)  C-9 (155.5) |
| 5 | - | 156.2 | - | - | - | - |
| 6 | 5.92 (1H, d; *J* = 1.80) | 94.9 | - | C-6 (94.9) | C-5 (156.2)  C-7 (156.5) | C-8 (94.2)  C-10 (99.5) |
| 7 | - | 156.5 |  |  | - | - |
| 8 | 5.85 (1H, d; *J* = 1.80) | 94.2 | - | C-8 (94.2) | C-9 (155.5) | C-6 (94.9)  C-10 (99.5) |
| 9 | - | 155.5 | - | - | - | - |
| 10 | - | 99.5 | - | - | - | - |
| 1’ | - | 130.9 | - | - | - | - |
| 2’ | 6.83 (1H, d; *J* = 1.20) | 113.9 |  | C-2’ (113.9) | C-3’ (144.9) | C-2 (81.5)  C-4’ (144.9)  C-6’ (118.7) |
| 3’ | - | 144.9 | - | - | - | - |
| 4’ | - | 144.9 | - | - | - | - |
| 5’ | 6.76 (1H, d; *J* = 8.00) | 114.7 | - | C-5’ (114.7) | C-4’ (144.9) | C-1’ (130.9)  C-3’ (144.9) |
| 6’ | 6.72 (1H, dd) | 118.7 | - | C-6’ (118.7) | C-5’ (114.7) | C-2 (81.5)  C-2’ (113.9)  C-4’ (144.9) |


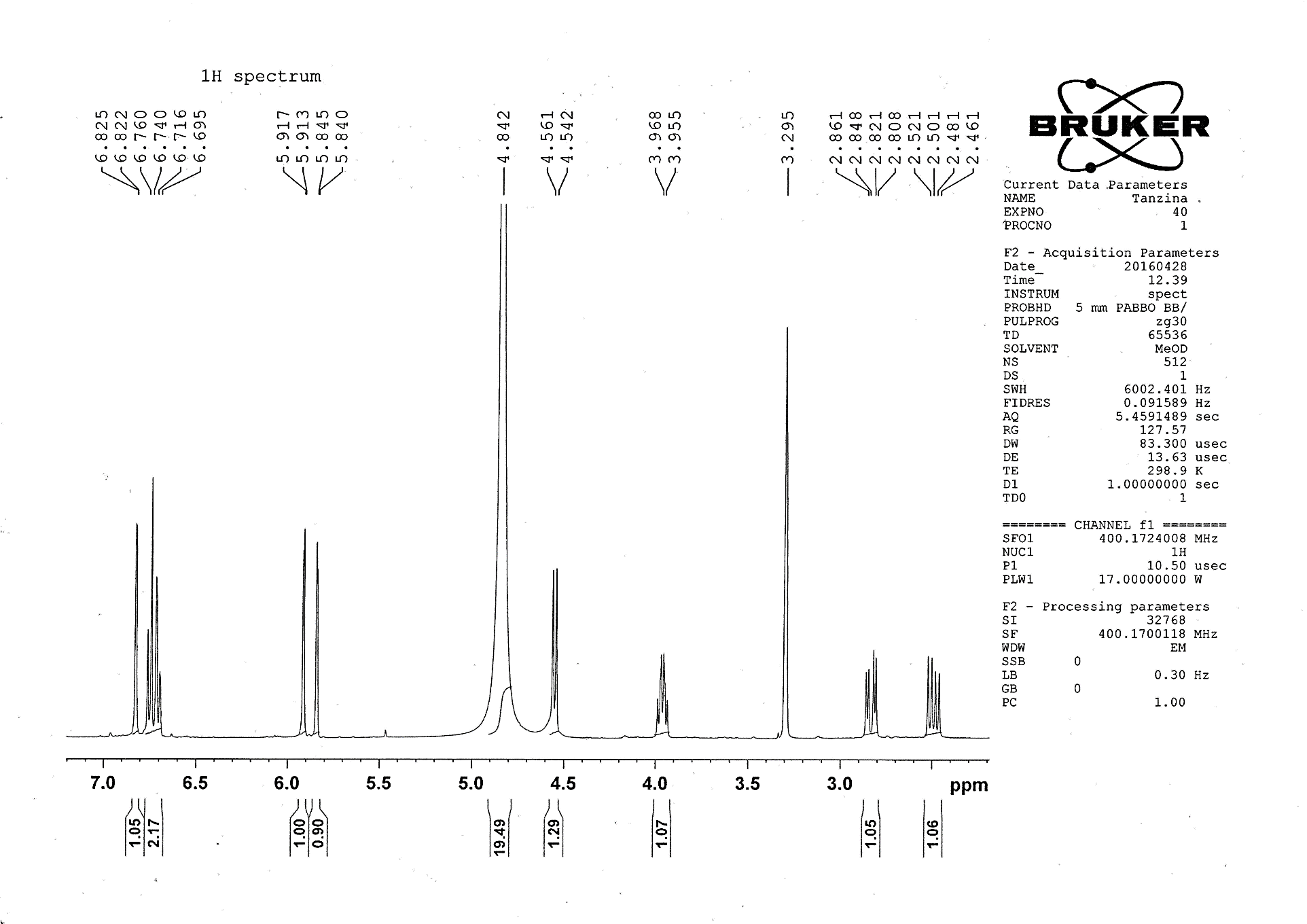


Fig S2. ^1^H NMR (400 MHz, CD_3_OD) spectrum of (+)-catechin.


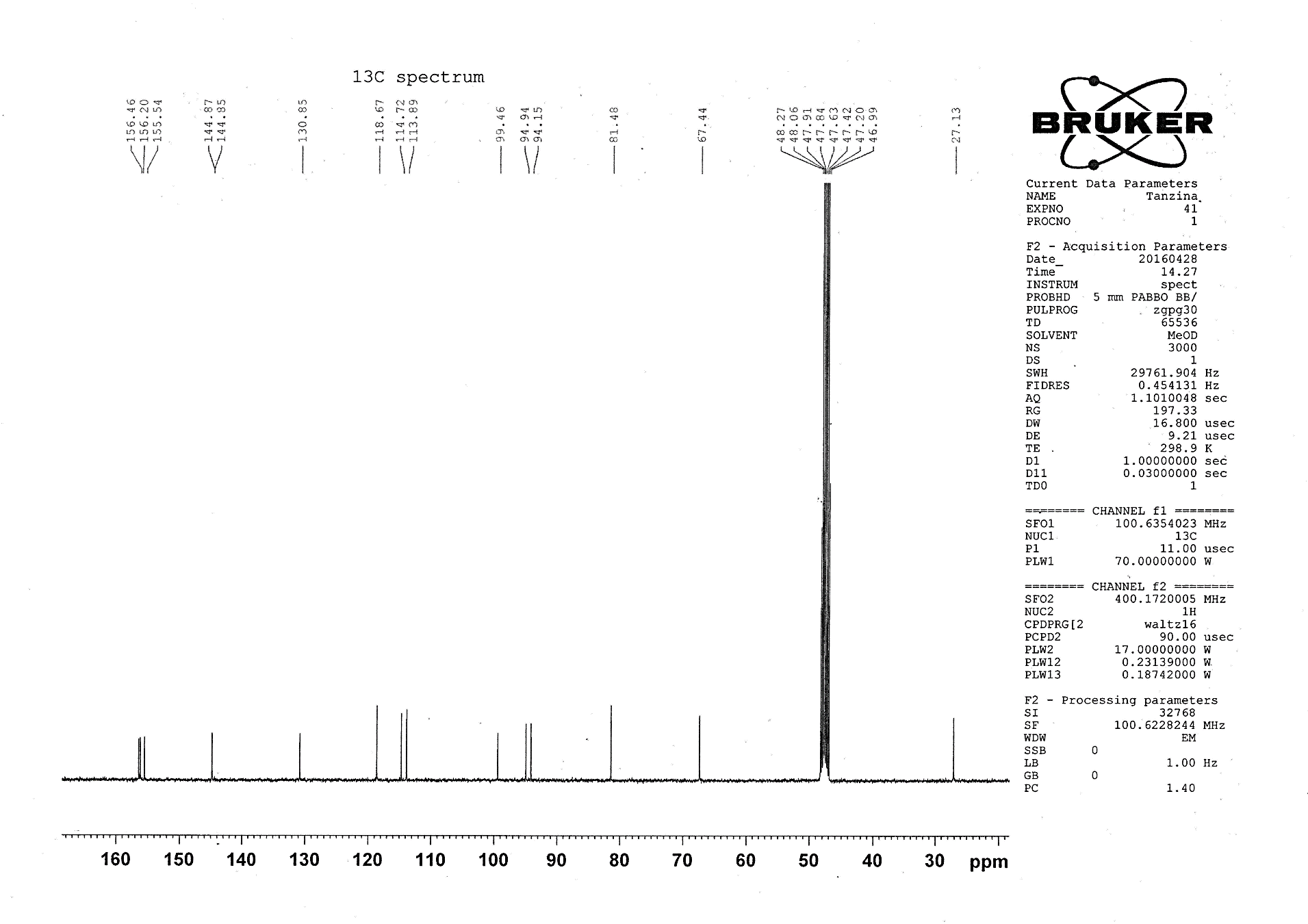


Fig S3. ^13^C NMR (100 MHz, CD_3_OD) spectrum of (+)-catechin.


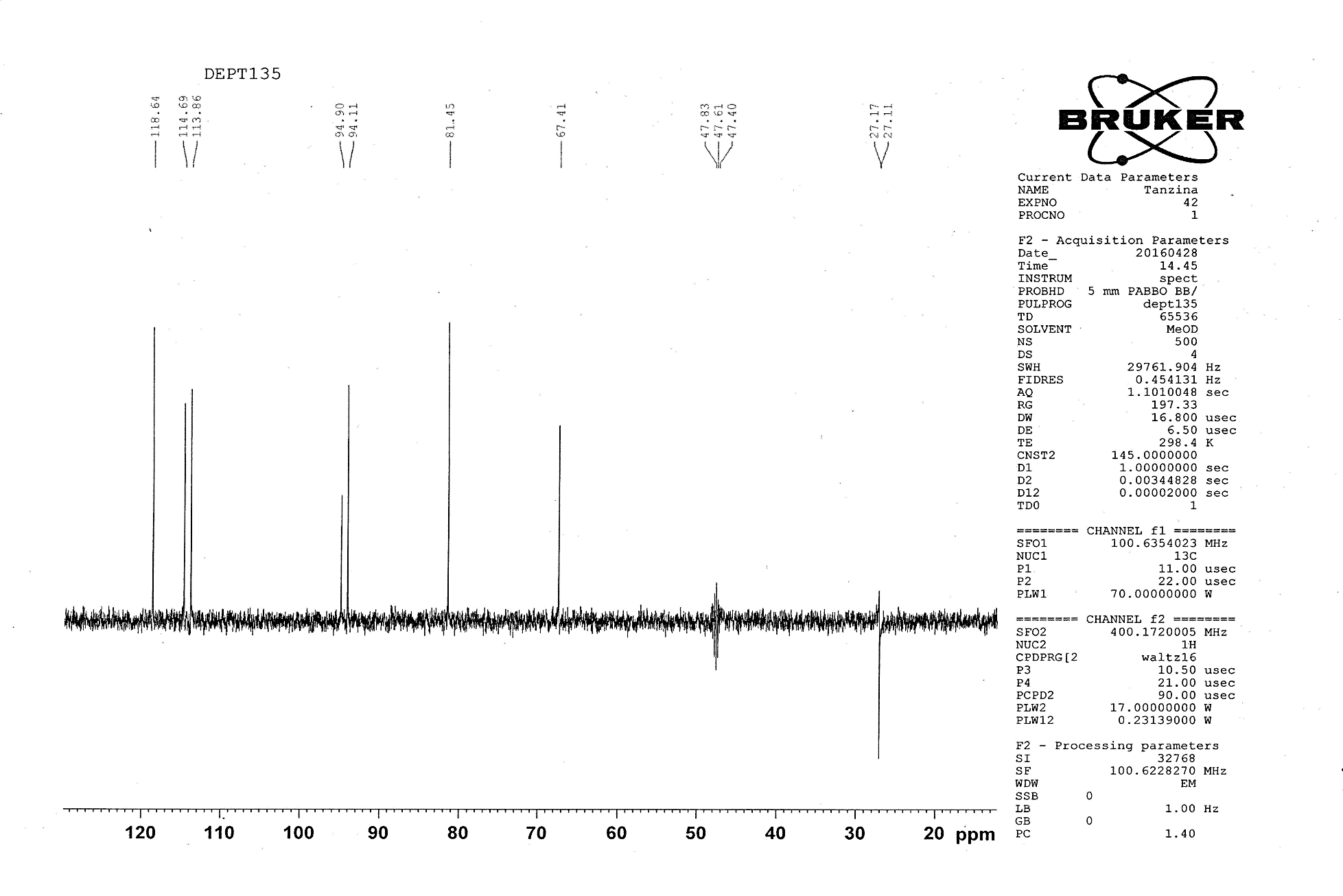


Fig S4. DEPT NMR (100 MHz, CD_3_OD) spectrum of (+)-catechin.


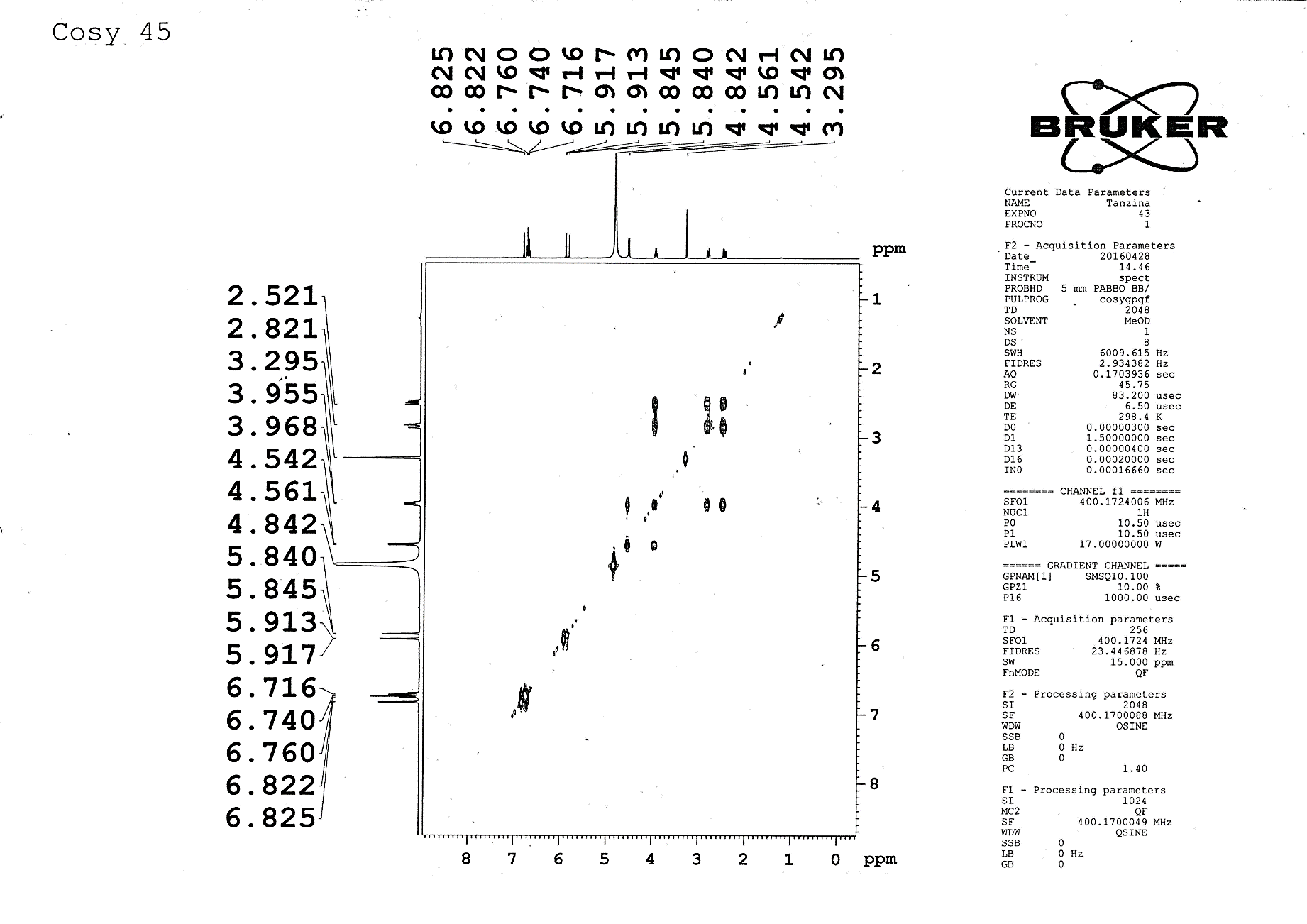


Fig S5. COSY NMR (400 MHz, CD_3_OD) spectrum of (+)-catechin.


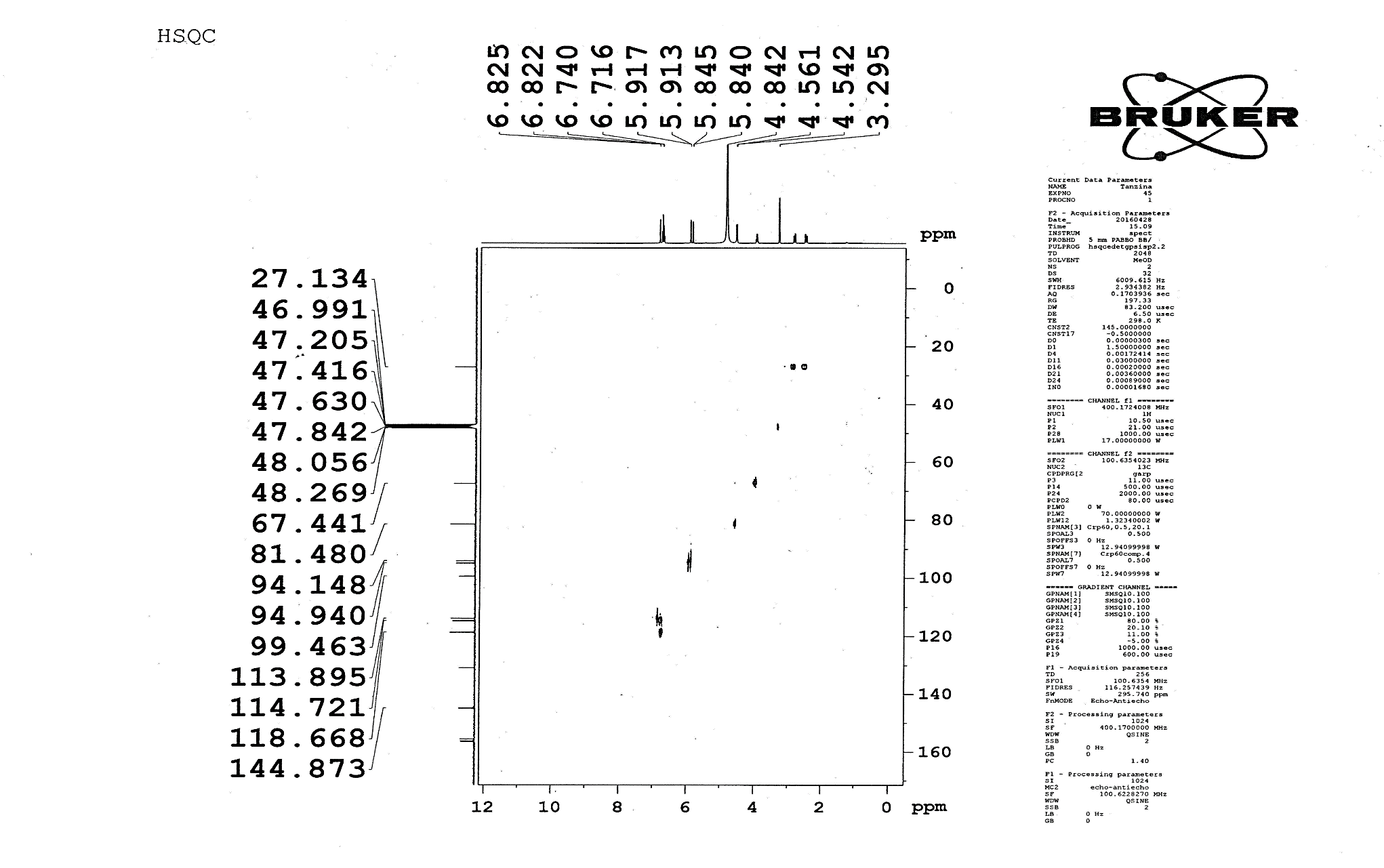


Fig S6. HSQC NMR spectrum of (+)-catechin.


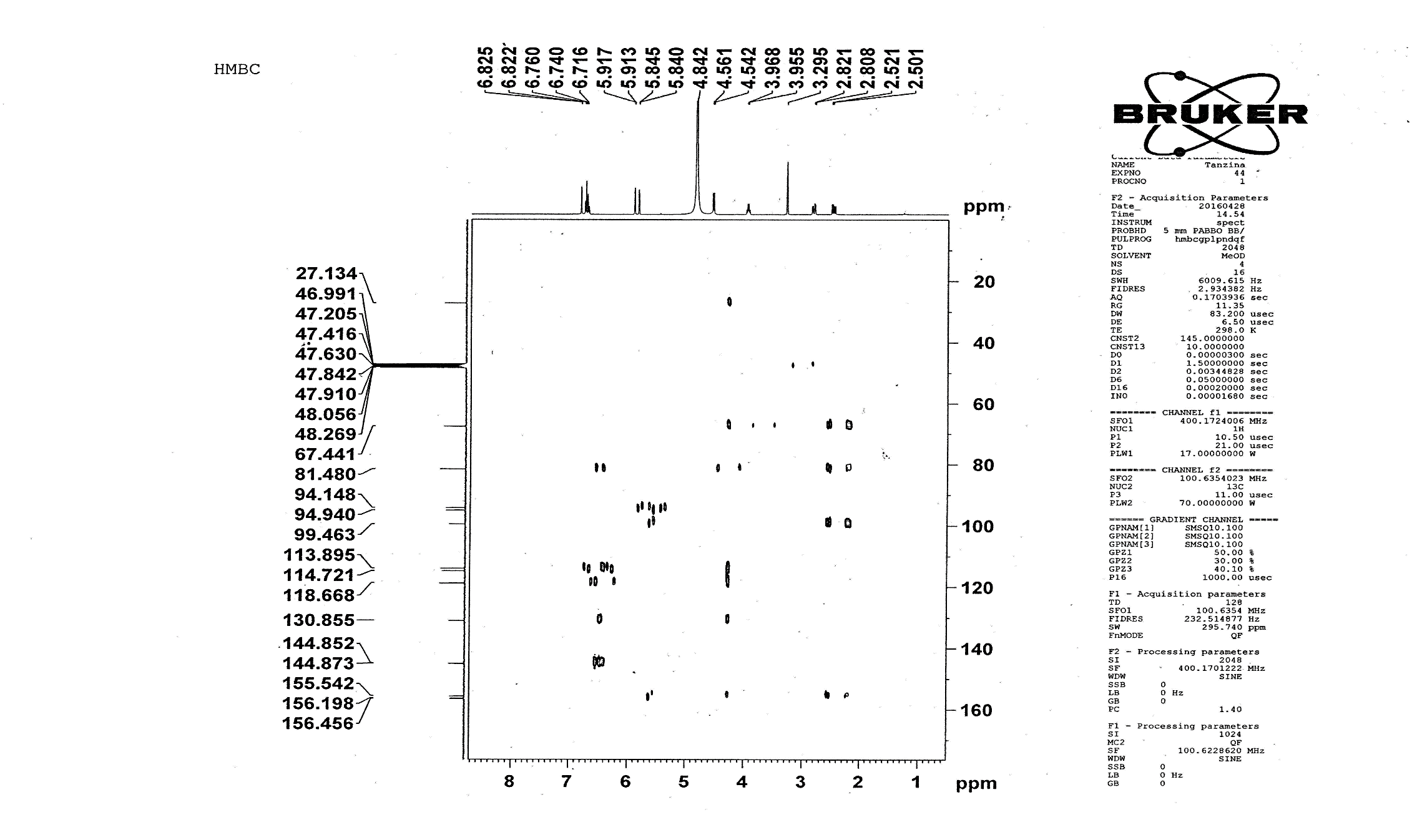


**Fig S7**. HMBC NMR spectrum of (+)-catechin.
